# Value configurations in sharing economy business models

**DOI:** 10.1007/s11846-020-00433-w

**Published:** 2021-01-20

**Authors:** Andreas Reuschl, Victor Tiberius, Matthias Filser, Yixin Qiu

**Affiliations:** 1 PricewaterhouseCoopers GmbH Wirtschaftsprüfungsgesellschaft, Querstraße 13, 04103 Leipzig, Germany; 2 Faculty of Economics and Social Sciences, University of Potsdam, August-Bebel-Str. 89, 14482 Potsdam, Germany; 3 School of Management and Law, ZHAW Zürich University of Applied Sciences, Gertrudstrasse 15, 8401 Winterthur, Switzerland; 4 LUT School of Business and Management, Lappeenranta University of Technology, Yliopistonkatu 34, 53850 Lappeenranta, Finland; 5 Chair of Strategic Management and Organization, University of Bayreuth, Prieserstraße 2, 95447 Bayreuth, Germany

**Keywords:** Sharing economy, Business model, Customization, Standardization, Property rights, Value creation, Value capture, Value configuration, L15, M10, M13

## Abstract

The sharing economy gains momentum and develops a major economic impact on traditional markets and firms. However, only rudimentary theoretical and empirical insights exist on how sharing networks, i.e., focal firms, shared goods providers and customers, create and capture value in their sharing-based business models. We conduct a qualitative study to find key differences in sharing-based business models that are decisive for their value configurations. Our results show that (1) customization versus standardization of shared goods and (2) the centralization versus particularization of property rights over the shared goods are two important dimensions to distinguish value configurations. A second, quantitative study confirms the visibility and relevance of these dimensions to customers. We discuss strategic options for focal firms to design value configurations regarding the two dimensions to optimize value creation and value capture in sharing networks. Firms can use this two-dimensional search grid to explore untapped opportunities in the sharing economy.

## Introduction

The sharing economy comprises all activities related to sharing or granting access to goods and services (Hamari et al. 2016). Sharing is organized in sharing networks. A focal firm manages the platform which connects the shared goods providers and customers. Sharing creates value by allowing customers to utilize products or services without acquiring ownership (Bardhi and Eckhardt 2012; Belk 2014; Hartl et al. 2016; Richter et al. 2015). The economic impact of sharing among actors (Zervas et al. 2017) alters the current mechanisms of value creation and value capture in business models as introduced by Richardson (2008) and Teece (2010). Sharing-based business models differ from traditional business models because value creation and value capture also are ‘shared’ among the sharing network members. Many sharing-based business models differ from current platform business models in that they match not only supply and demand for digital but also physical goods that require’real world’ logistics. However, some sharing-based business models such as sharing WI-FI connections are purely digital.

Due to its practical relevance, research on the sharing economy has gained momentum (Belk 2014; Filser et al. 2020a; Hamari et al. 2016; Hartl et al. 2016; Kraus et al. 2020a; Moehlmann 2015; Oskam and Boswijk 2016). It focuses on successful sharing-based business models like accommodation providers such as Airbnb (Gassmann et al. 2021; Oskam and Boswijk 2016; Zervas et al. 2017), coworking space providers such as WeWork (Bouncken et al. 2018, 2020a, b; Vidaillet and Bousalham 2020), transportation service providers such as Uber (Cohen and Kietzmann 2014), car sharing providers such as Car2Go or ShareNow (Bardhi and Eckhardt 2012; Cohen and Kietzmann 2014; Wallsten 2015), the sharing of WI-FI connections, computers, services, food and other goods (Belk 2014), and many others (Geissinger et al. 2020). As an example for B2B sharing, Apple and Dell ‘share’ the production facilities of Foxconn (Chan et al. 2013).

Information and communication technologies provide the basis for these business models (Belk 2014; Fjeldstad et al. 2012). Apart from common web-based technologies, the currently evolving ‘Internet of Things’ (IoT) offers opportunities for new business models (Brettel et al. 2014; Lee et al. 2015; Metallo et al. 2018). It integrates machines and production facilities into the sharing economy (Bardhi and Eckhardt 2012; Belk 2014). Beyond mere profit maximization, offering sharing-based products or services can also be seen as a societal engagement. In particular, sharing addresses sustainability concerns (Curtis and Lehner 2019; Govindan et al. 2020; Hamari et al. 2016; Liu and Chen 2020; Pies et al. 2020; Ponce et al. 2018; Pouri and Hilty 2018).

Despite the current research intensity regarding the sharing economy, we still lack an in-depth understanding of business models in the sharing economy (Belk 2014; Cohen and Kietzmann 2014; Hartl et al. 2016; Pies et al. 2020) and, in particular, of the mechanisms of value creation and value capture configurations. Therefore, our research goal is to identify sharing-specific antecedents of value creation and value capture. To achieve this research goal, we conduct a qualitative study on 18 sharing-based cases (study 1).

Our results reveal two independent dimensions of integrated value networks: (1) the degree of customization or standardization of the shared goods and (2) the distribution of property rights over key resources, i.e. their centralization or particularization, especially a focal firm’s degree of control over the shared goods. A quantitative study (study 2) among (potential) customers confirms the public visibility of these two dimensions. Based on these insights, we discuss the strategic options a focal firm has to shape optimal value configurations by a suitable arrangement of the two dimensions. These two independent dimensions provide a grid which allows analyzing the value configurations and thus the strategic positions of firms in the sharing economy. Researchers and firms can use this grid for a strategic analysis that explores not yet occupied market spaces. Focal firms can create novel business models and platforms within these dimensions. Our research contributes to both the sharing economy literature and research on business models and their value creation and value capture mechanisms. The identification of the two dimensions provides a first guidance for practitioners who create and researchers who investigate sharing networks. Our results help researchers and practitioners to better understand how firms can achieve and enhance the advantages of the sharing economy.

## Theoretical background

### Sharing economy

No commonly accepted definition for the sharing economy exists as it is still a young phenomenon. Current research streams focus on framing the concept of the sharing economy (Arvidsson 2018; Bardhi & Eckhardt 2012; Belk 2014; Cheng 2016; Martin 2016), reasons and motivation for participation (Davidson et al. 2018; Hamari et al. 2016; Möhlmann 2015) and governing mechanisms (Ert et al. 2016). Recently, emerging research streams with in-depth sharing economy research questions on the internationalization process (Parente et al. 2018), industry specifics for example for apparel (Park and Armstrong 2017), hotel business (Zervas et al. 2017), or mobility (Cohen and Kietzmann 2014) outline the growing maturity and acceptance of the research field. The sharing economy can even be viewed as an entrepreneurial ecosystem of its own kind as it attracts new providers of shared goods (Liguori et al. 2019).

As we focus our research on sharing-based business models, we follow Hamari et al. (2016, p. 2047) and define the sharing economy as “peer-to-peer-based activit[ies] of obtaining, giving, or sharing the access to goods and services, coordinated through community-based online services”. This definition allows us to include all forms of web-based sharing activities including incumbents that run sharing economy-like business models (Belk 2014; de Lange and Valliere 2020; Hamari et al. 2016). Web-based connectivity enables consumers to connect, exchange information, and coordinate sharing activities without restrictions of time and space, resulting in the development of novel business models (Afuah 2003). Web-based information and communication technologies enable enhanced value creation, as goods and services are shared only for the time needed (Belk 2014; Hamari et al. 2016). Therefore, the internet integrates or even generates markets, links their participants across boundaries and contributes to the emergence of globally unified markets (Pohjola 2002).

The collaborative consumption of goods and services (Hartl et al. 2016) changes consumers’ attitudes towards property and ownership. Customers focus on distinct access rights for using goods and services for the limited time span when their utilization rather than acquiring ownership or long-term property rights are needed (Bardhi and Eckhardt 2012; Belk 2014; Hartl et al. 2016). Ownership can especially be replaced by permanent access when customers are loyal to the sharing provider (Akhmedova and Marimon 2020; Jia et al. 2020). This trend also impacts B2B relations as technical improvements allow to ‘share’ production capacities and thus to integrate production capacities into sharing systems (Belk 2014; Brettel et al. 2014; Lee et al. 2015).

### Business models, value creation and value capture

Even though business models have been in existence since mankind discovered trading (Teece 2010), the emergence of e-commerce and other internet-based products and services has massively intensified research on business models (Amit and Zott 2001, 2012; Demil et al. 2015; Foss and Saebi 2017; George and Bock 2011; Osterwalder 2004; Zott et al. 2011). For business models, many definitions exist (Zott et al. 2011). Especially in entrepreneurship, business models have become a popular perspective (Ferreira et al. 2019). Business models can be seen as architectures (Teece 2010; Timmers, 1998), blueprints (Osterwalder et al. 2005), designs (George and Bock 2011; Teece 2010), frameworks (Chesbrough and Rosenbloom 2002), or representations (Morris et al. 2005) of “how firms do business” (Zott et al. 2011, p. 1037). Business models comprise several components (Zott et al. 2011), dimensions (Osterwalder & Pigneur 2010) or elements (Baden-Fuller and Mangematin 2013). For example, Amit and Zott (2001, 2010, 2012) distinguish content, structure and governance (Amit and Zott 2001, 2010, 2012). Content depicts the activities performed in the activity system, including the exchange of products, services and information between the various network partners as well as the capabilities required to enable the exchange. Structure describes the linkages and the sequencing of these activities, considering size, flexibility and adaptability of networks. Governance describes who performs which activities as well as the locus and nature of control of transactions within the activity system. Another structure is suggested by George and Bock (2011) who, based on a survey among practitioners, distinguish a resource, transaction and value structure. Especially the business model canvas, as suggested by Osterwalder (2010) and Osterwalder and Pigneur (2010), is well established among both scholars and managers. It defines nine dimensions of the business model structure: key partners, key activities, key resources, the value proposition, customer relationships, channels, customer segments, the cost structure, and revenue streams.

In contrast to traditional strategic management which focuses on competitors, the business model approach focuses more strongly on customers (Demil et al. 2015; Zott et al. 2011). However, superior configurations of business models can generate competitive advantages (Markides and Charitou 2004). But with its strong customer orientation, a business model’s predominant dimension is its value proposition (Chesbrough and Rosenbloom 2002; Morris et al. 2005). More specifically, the firm has to define how it will create (and deliver) this offered value to the customers. Business models in the sharing economy do not necessarily have to offer innovative content but often only the flexibility or the details of content increase in comparison to traditional business models. The predominant role of the value proposition becomes particularly apparent in Teece’s (2010, p. 172) definition of business models as a “design or architecture of the value creation, delivery, and capture mechanisms”. Therefore, business models refer to the creation and capture of value from the combination of activities (e.g., IT and operations) into solutions, especially when acting in networks (Bouncken and Fredrich 2016). In exchange for the expected or experienced value (i.e., use or benefit) of the firm’s offering, the customers pay for it generating revenue and profit for the firm. Therefore, for a firm to maximize its extent of value capture, it has to offer a value proposition in a way the customer is willing to pay most. These notions are not only valid for individual firms in a market but also for decentralized business models in the shared economy. They relate to the network of the focal firm, key partners and customers because value creation is dependent on the firm’s resources and external property. Value delivery depends on providing these external goods or services to the customers. The value captured has to be split among the participants. Activities exceed the mere use of technologies (Chesbrough 2007) and cross the boundaries of single firms that are often embedded in networks (Amit and Zott 2001). Thus, the business model approach is well suited for explaining value creation in the sharing economy. Sharing economy business models can be based on platform business models (Clauss et al. 2019; Muzellec et al. 2015; Täuscher and Laudien 2018). The match between supply and demand of shared goods occurs on platforms for which technology plays a constituting role as facilitator for self-linking processes among participants (Thuong and Monideepa 2009). However, while regular platforms deliver digital goods which hardly cause any storage or delivery costs or waiting time for customers, many sharing-oriented business models involve the storage and time-consuming delivery of physical goods that can not only be coordinated digitally.

Business model innovation is associated with the agile and radical redesign of extant business models that is based on dynamic capabilities (Heider et al. 2020; Semke and Tiberius 2020) and aims at fostering growth, firm performance, and the development of a competitive advantage (Amit and Zott 2012; Bouncken et al. 2016; Bouncken et al. 2019; Brand et al. 2019; Breier et al. 2021; Kraus et al. 2020c; Tiberius et al. 2020a). Apart from radical business model innovation, firms also implement incremental business model reconfigurations (Clauss et al. 2020). In both respects, sharing-based business models of both start-ups and incumbents have to be considered as innovative business models. Innovative business models as novel combinations of their components result in value creation, delivery, and capture forms that are new to a market (Teece 2010). Unique or novel value propositions allow new ways of value creation through new products or services and of value capture by, for example, new payment models such as membership fees or transaction-based payment.

Firms that do not yet participate in the sharing economy but consider doing so can add an innovative business model to their current one(s). For example, while car manufacturers Daimler and BMW, which usually focus on selling cars, have been offering their car sharing providers car2go and DriveNow (that have recently merged to their mutual provider Share Now) for several years, Volkswagen is only about to enter the sharing economy. Apart from sharing the firm’s own goods, firms can also cooperate with several partners, which contribute complementary goods or services to increase heterogeneity and extend the activity and customer base, like in the case of Flinkster, also a car sharing provider, which integrates further complementary transportation services into the sharing network and to provide a comprehensive mobility portfolio. Flinkster is a remarkable role model for sharing economy business models that considerably extend firms’ traditional scope of action by providing the opportunity to access new markets and customers. Other firms like Share Now use social networks to access their customer base, create lock-in effects and improve marketing. This form of horizontal integration offers additional synergetic advantages. For example, embedding car sharing communities in or connecting them with social networks can optimize occupancy, create marketing effects or facilitate the development of additional services.

## Methodology

### Mixed method approach

**Fig. 1:**
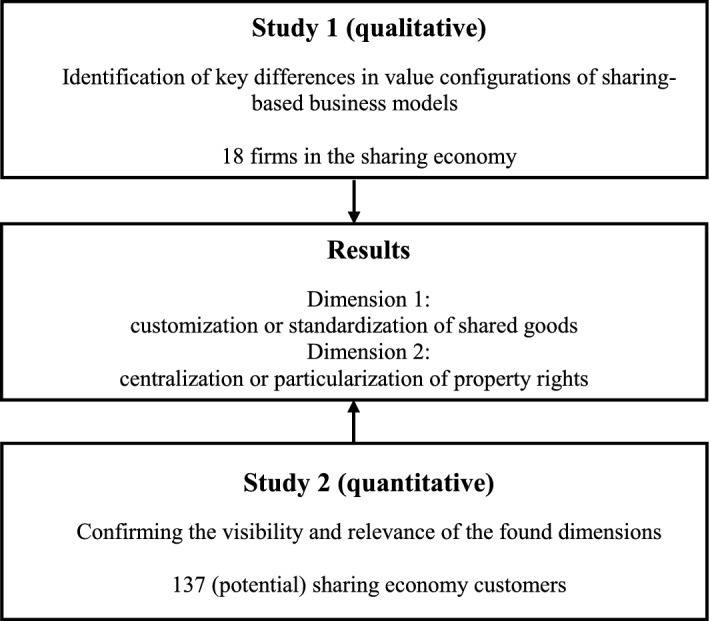
Research methodology

We chose a mixed method approach comprising a qualitative and a quantitative study (Creswell 2003) (Fig. 1). The qualitative approach aims at gaining in-depth insights about rather unexplored phenomena to generate rather than validate propositions based on small samples (Eisenhardt and Graebner 2007), while the quantitative approach tries to validate already existing hypotheses based on larger samples. For our research question, no hypotheses exist yet. Therefore, focusing on theory building, our research is inductive in nature (Yin 2009) and we do not use predefined propositions from literature. With our second study, we aim at validating the prior findings.

### Study 1

In the first step, we followed the case study literature (Eisenhardt, 1989; Eisenhardt and Graebner 2007) and selected 18 case firms. To ensure that the cases match our research focus, we selected for-profit case firms that participate in the sharing economy or employ business models that are based on or related to the idea of sharing irrespective of the specific industry. A short overview over the case firms is given in Table 1.

**Table 1:** Case examples

Case firm	Founding year	Website	Description
Airbnb	2008	www.airbnb.com	Airbnb offers a platform for a peer–to–peer network that enables users to share their accommodation
Car2Go	2008	www.car2go.com	Car2Go (Daimler AG) offers a car sharing network with cars from Smart and Mercedes
DriveNow	2011	www.drive-now.com	DriveNow offers a car sharing network with cars from BMW and Mini
Enterprise	1957	www.enterprise.com	Founded in the USA, Enterprise is the largest American car rental company. The business model for renting cars is similar to the idea of car sharing
Europcar	1949	www.europcar.com	Europcar was founded in France and is a major European car rental company. The business model for renting cars is similar to the idea of car sharing
Flinkster	2009	www.flinkster.de	Flinkster belongs to the German railway company Deutsche Bahn and offers car sharing and car rental services
Fon	2005	www.fon.com	Fon operates a sharing economy approach for Wi–Fi networks
Getaround	2009	www.getaround.com	Getaround provides a peer–2–peer car sharing network
Greenwheels	1995	www.greenwheels.com	Greenwheels is the largest car sharing network in the Netherlands
Hapimag	1963	www.hapimag.com	Members of Hapimag are shareholders, invest in vacation properties and are entitled to use these
HILTI	1941	www.hilti.com	Hilti offers a rental model for premium tools
Lending–Club	2006	www.lendingclub.com	LendingClub is a US–based peer–to–peer money lending network
MyHammer	1999	www.my-hammer.de	Myhammer offers an online network that mediates (handicraft) services
Turo	2009	www.turo.com	Turo (formerly RelayRides) provides a peer–2–peer car sharing network
Sixt	1912	www.sixt.com	Sixt was founded in Germany and is a major European car rental company. The business model for renting cars is similar to the idea of car sharing
TaskRabbit	2008	www.taskrabbit.com	TaskRabbit offers a network for all kinds of services on–demand
UBER	2009	www.uber.com	Uber offers an application for a peer–to–peer network for taxi services
Zaarly	2011	www.zaarly.com	Zaarly provides a network where users create own stores and offer goods or services to users

We collected publicly available data from the selected firms from internal sources such as webpages, brochures and annual reports and from external sources such as media coverage. Drawing on these archival data, we analyzed the case firms’ value configurations. In case of missing information, we contacted the firms to complete our data. As suggested by Eisenhardt (1989) and Ram and Trehan (2009), we applied an iterative data analysis process. First, we condensed the available information and created write-ups for each individual case. These write-ups were analyzed based on an open coding procedure. Next, we compared the individual case results in a cross-case analysis (Eisenhardt 1989). Third, we re-analyzed the write-ups and applied a numerical weighting ranging from 1 to 10 on the identified categories. Two researchers independently carried out the iterative process of analyzing the case data and four researchers weighted the identified categories to enhance rigidity and to ensure consistency of the findings.

**Fig. 2:**
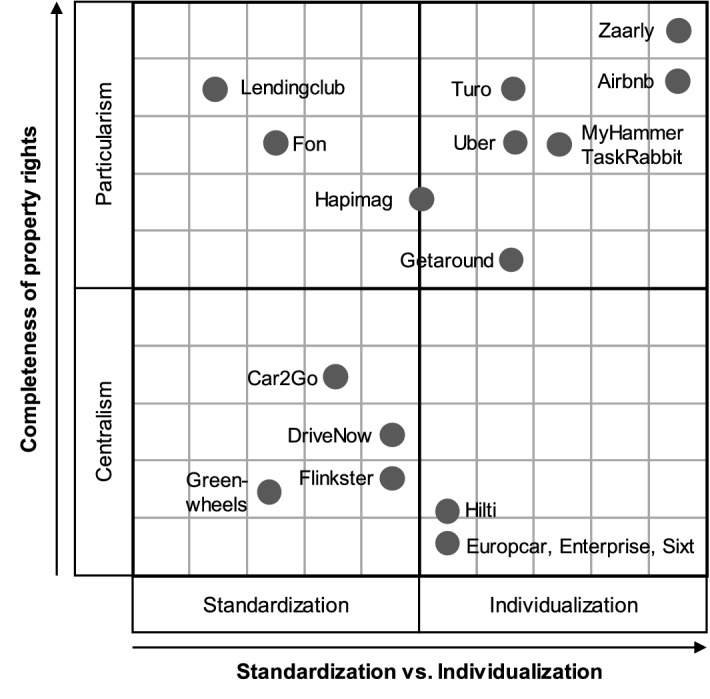
Value configuration positions of the case firms, rated by researchers

Our findings show that, from a value configuration perspective, two predominant dimensions seem to apply to all cases: (1) The spectrum of customization to standardization of the value proposition and (2) the spectrum of centralized to particularized property rights, i.e., their distribution, over key resources. These two independent dimensions allow analyzing the value configurations and thus the strategic positions of firms in the sharing economy. Figure 2 illustrates the two-dimensional framework and the positioning of the case firms in it, as rated by the researchers.

### Study 2

In the second step, we conducted a survey among 137 undergraduate students in management classes. In line with Gupta et al. (2019), we decided to survey undergraduate students because they are highly accustomed to digital business models and have a high propensity to participate in the sharing economy.

Our short questionnaire included questions on the respondent’s age, course of study, gender and a list of the case study firms to be rated on the two value configuration dimensions. We asked the students to indicate if they have already used services from the firms and to rate the experienced property rights (scale from 1 to 5, 1 = fully owned and controlled by provider, 5 = highly decentralized, provider only owns platform) and customization (scale from 1 to 5, 1 = highly standardized, “one fits all offering”, 5 = highly customized, divers and unique offering).

We received 68 responses (49.6%) from students at the age between 18 and 30, 29.4% male and 70.6% female respondents. A majority of 88.2% of the respondents indicated that they have already used the services of at least one case firm. One student already used services from seven of the case study firms, the average is 2.43.

**Fig. 3:**
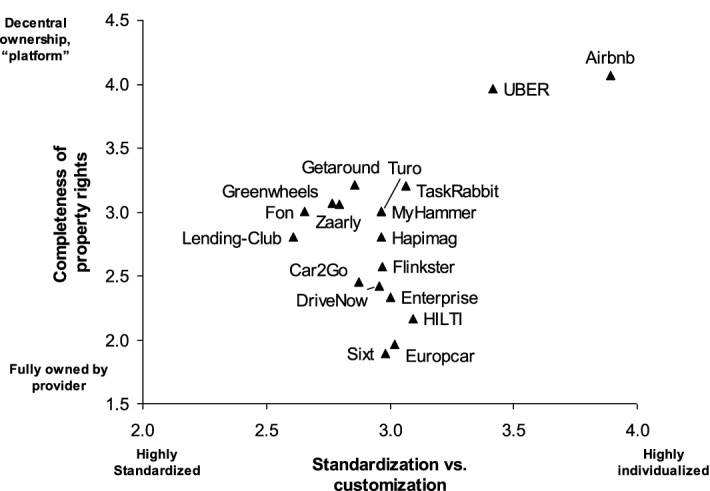
Value configuration positions of the case firms

The results of our survey highlight the public visibility of the value configuration regarding customization and standardization and distribution property rights to customers. Figure 3 illustrates the answers of our 68 survey participants as mean values.

Highly successful sharing economy companies like Uber and Airbnb are perceived as platforms that organize the utilization of highly customized goods with decentral ownership. In contrast, strong and successful incumbents like Sixt, Europcar or Enterprise are known to offer goods or services that are fully owned by the firm. Consistently, sharing business models of incumbents (Flinkster, ShareNow) are located between the two extremes.

However, the platform providers Turo, Zaarly, MyHammer and Taskrabbit are not perceived as particularized and customized as we suggested in Fig. 2. A possible and likely explanation for the unexpected localization of the case firms is that students do not use the offerings of these firms since they are not the right target group. For example, MyHammer and Taskrabbit offer mainly handyman services, Zaarly is not active in Germany and Turo has only very limited availability in Germany.

**Fig. 4:**
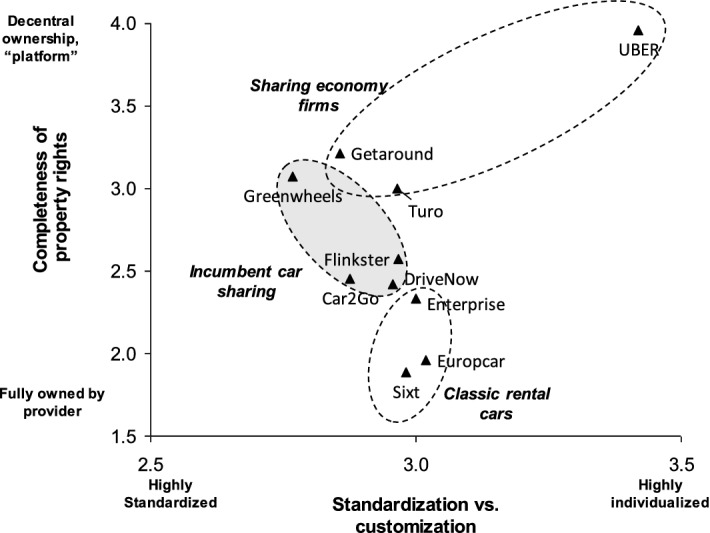
Value configuration positions of the carsharing case firms

Focusing on mobility suppliers provides further insights on current value configurations. The business models of the case study firms in Fig. 4 center on mobility services. However, sharing economy value configurations offer new design themes for the related business models. Uber represents a role model for these novel design themes. Its business model completely concentrates on matching the demand and supply for mobility services. Sharing economy firms like Getaround or Turo follow this business model but also offer cars without chauffeurs. In contrast, classic rental car incumbents fully control the supply side and compete for the existing demand. Car sharing ventures of incumbents take a mixed approach as they have full ownership over the offering but they have a technology enabled offering like a sharing economy firm.

## Findings

### Customization and standardization

The content in the sharing economy is often less standardized than in traditional business models. For example, while hotel rooms of major hotel chains resemble one another, Airbnb offers a huge variety of private ‘hotel rooms’, following the ongoing trend for customization. However, our cases indicate that shared goods can be highly customized or standardized. Customization relates to a market, customer and quality orientation while standardization follows the logic of industrialization or mass production (Sundbo 2002), and therefore economies of scale. The general tendency towards standardization can be due to price pressure caused by intense competition (Sundbo 2002). However, standardization can also be a deliberate strategy, namely Porter’s (1987) generic competitive strategy of cost leadership while customization relates to a differentiation strategy. Customers who are interested in customization have hedonistic goals, strive for uniqueness and experience a higher perceived control, satisfaction but also a higher perceived risk while customer who are more oriented towards standardization have utilitarian goals and are more interested in saving time and money (Ding and Keh 2016). Therefore, both strategies meet different customer needs and preferences. Sharing economy firms should identify and target their preferred market segments and target groups (Lutz and Newlands 2018).

From our case firms, former Car2Go initially offered only one standardized car that met the average users’ expectations and a set of specific car sharing requirements. Using shared cars is characterized by user’s low emotional involvement. Without the intention to acquire a car, users are not interested in customizing it.

In contrast, highly customized goods allow a special emotional experience. For example, Airbnb provides “boutique-style” accommodation, i.e. rooms, apartments or houses, with their floor plans and furnishings, are unique. The users can book the accommodation that best fits his or her needs and preferences. The interior or the social interaction with the provider of the accommodation or other users can create special and unique experiences. This customized experience is a key value proposition. To allow greater customization, a focal firm such as Airbnb has to provide mechanisms for customers to choose an adequate offering. Unstandardized solutions demand more trust building and access to ratings and feedback from users. Thus, sharing economy firms such as Airbnb that offer unstandardized goods require more detailed information for users. The empirical study of Gupta et al. (2019) shows that the propensity of sharing products (supply and demand) decreases with the level of intimacy. Therefore, the degree of customization should be investigated further to identify emotional barriers that might impede the attractiveness of shared products.

### Property rights

Business models in the sharing economy offer governance mechanisms allocating responsibilities for activities (Zott and Amit 2010). The focal firm, network partners, or consumers carry out activities to co-produce value. Our cases show that the governance structure exceeds mere task allocation as it also covers property rights. Property rights grant the bundle of rights (Coase, 1960) to exploit and alienate a resource (Alchian, 1965; Asher et al. 2005; Demsetz, 1967). They enable the owner to generate high rents (Foss and Foss 2005) and therefore capture value, especially when protection mechanisms are used (Teixeira and Ferreira 2019).

At first sight, it might be striking that property rights form an important dimension of business model value configurations, as sharing means waiving ownership and only striving for access to goods. However, this is only true from the customers’ perspective. Shared goods are not ownerless (res nullius), public (i.e., everybody’s) property, and usually not even common (i.e., jointly owned) property (Bromley, 1991). Rather, only the right to *use* the good is shared, whereas other partial property rights, i.e., the right to earn income from the good, the right to ownership cessation, and the right to enforce property rights (Coase, 1960), belong to specific actors in the sharing network. These property rights which exceed mere usage are not shared.

The distribution of property rights refers to the platform and the shared goods. This influences who in a network consisting of the focal firm, key partners, and customers is able to make property-related decisions (Mumdziev and Windsperger 2011) and to capture and guard created value. Using, designing, organizing, and controlling the sharing processes requires the consideration of the distribution of property rights among key partners. Property rights can be concentrated on focal firms or spread on diverse individual owners. Even without full ownership, focal firms can have control over design and shape of goods based on contractual property rights. The centralization of property rights has major implications for design, functionality, organization and maintenance of shared goods, affecting long-time use and interests. Cesinger et al. (2016) demonstrate the importance of ownership and non-financial values in family firms. Particularized property rights—not to be confused with partitioned property rights (Alchian, 1965; Ostrom, 1990)—increase the probability of private owners sharing goods. Besides economic returns, though, they will also care for socio-emotional issues, conflicts and misuse. The experience and satisfaction with sharing is influencing their willingness to continue sharing their property and interacting with users (Moehlmann 2015).

Car rentals like Europcar, Sixt, or Enterprise completely own the property rights of their standardized products and allow customers to use vehicles for a fee. Customization is only possible in terms of providing additional services or cars with predefined features. Based on the principle of crowdfunding (Kraus et al. 2016), LendingClub organizes a peer-to-peer network that allows members granting and obtaining loans. This illustrates an integrated value network where individuals (high particularism) own and give temporal property rights for a highly standardized content (money). Similarly, Fon organizes a community of individuals that share the standardized content ‘access to WI-FI’. Individuals retain property rights over their WI-FI access (high particularism). Airbnb exemplifies a sharing network where individuals contribute customized content to a network without transferring property rights. Airbnb provides the constituting technology that allows network members to initiate contacts and perform business transactions. Thus, Airbnb achieves a position where highly customized content of a large variety of users is integrated into a network that creates value. Zaarly and TaskRabbit also strive to achieve such a position. They provide networks where individuals can offer or demand any kind of service or product to or from other members. Zaarly and TaskRabbit do not gain property rights over the services, they provide the technology to organize networks that allow individuals to act entrepreneurially (Schmengler and Kraus 2010). BMW’s and Daimler’s car sharing provider ShareNow offers their own products. Providers retain property rights at the cost of customization. Car sharing platform providers like Turo or Greenwheels waive property rights for achieving higher levels of customization. Belonging to the German railway company Deutsche Bahn, Flinkster offers a variety of cars and vehicles from all major producers and holds the property rights over them. Thus, Flinkster offers a mobility concept that comprises train rides and different cars that meet individual demands. A special case between car sharing platform providers and company bound car sharing is Getaround which offers a network for carsharing like Turo or Greenwheels. However, network members who want to bring in their vehicles have to install a special corporate software and pay monthly fees. Thereby, Getaround achieves at least a partial property right over the cars of its users. This software is part of the constituting technology that enables transactions in the activity system of Getaround.

Therefore, at one extreme, a focal firm has complete property rights over goods, allowing for maximal rigor of influence. In this case, the firm only considers its own interests in the design and coordination of goods. At the other extreme, a focal firm is only a facilitator for sharing. In this case, further factors like specific business logics and socio-emotional interests of private individuals affect sharing dynamics. Particularized ownership demands complex mechanisms for coordination and control of interaction systems and governance solutions to deal with multiple motives of diverse owners.

## Discussion

The value configuration of a focal firm in the sharing economy, i.e., the characteristics of value creation and value capture, can be defined along the dimensions of customization and individualization on the one side and centralization or particularization of property rights over shared goods on the other side. Our first study revealed these independent dimensions as crucial differences in business models that are decisive for the value configurations in the sharing economy. The second study confirmed their significance from a customer perspective. A strategic analysis of these two value configuration dimensions of sharing-based business models can reveal spaces that are not occupied in the market yet. Therefore, the two-dimensional search grid allows for the exploration of innovative and profitable strategic alternatives for untapped future business opportunities (Filser et al. 2020b; Tiberius 2019; Tiberius et al. 2020b). Focal firms can create novel business models and platforms within these dimensions.

The first dimension—customization or standardization—relates to the value creation in sharing-based business models as it addresses customers’ value expectations ranging from economic, functional, socio-emotional to epistemic values (Sheth et al. 1991). This dimension thus relates to an external perspective of the value configuration. Customization means a better fit with the customers’ needs and preferences. Unlike the “one fits all” approach of standardization, for customization many different forms of the shared good exist and customers can choose the prefered one and, therefore, the selected good generates a higher value for the customer compared to a standardized one. However, also standardized products can generate value by being less costly due to economies of scale. The trade-off between customized and standardized shared goods relates to Porter’s (1987) generic competitive strategies of differentiation and cost leadership. For both target groups—customers who want unique goods or customers who want to save money—market segments exist. The internet decreases information asymmetries through comparison possibilities. Thus, quality becomes a decisive feature for the customer demand and for achieving a competitive advantage. However, the concept of quality expands in the context of the sharing economy. While traditional criteria such as performance, data security and processing speed maintain their importance, the uptime and uniqueness of goods are important for shared goods. As most focal firms offering shared goods operate on two-sided markets (Muzellec et al. 2015), not only the lender but also the supplier, both being the focal firm’s customers, have to be included in the value creation considerations. For lenders, customization is the normal situation since he or she owns a unique good for sharing. Only for large lenders who share several goods, standardization can be a cost-saving issue.

The second dimension—centralized or particularized property rights—represents an important internal perspective, as it strongly relates to the necessary governance mechanisms to manage the processes, transactions, and the determination of both value creation and value capture in the business model of a focal firm. The right to command resources defines how value is generated through their use (Claessens and Laeven 2003; Jandhyala 2013; Keay and Metcalf 2011). A focal firm having full property rights over the platform and the shared goods is able to define the value proposition and design of the platform, thereby shaping the characteristics and extent of value creation. A focal firm with centralized property rights can also capture a large proportion of the created value due to their larger sphere of influence compared with a focal firm which owns only the platform but not the shared goods. Our case studies indicate that centralized property rights that are with the focal firm have a dominant coordination function in the business model. Yet, centralized property rights must enable self-regulated coordination among users. Thus, decentralized and self-regulated coordination among users stands on the shoulders of formal governance and can build additional value. The properties of the two found dimensions are summarized in Table 3.

## Conclusion

Our paper uses theoretical considerations and case examples to analyze value configurations in the sharing-based business models, i.e., the design of value creation and value capture systems. Our results show that focal firms in the sharing economy create sharing networks with varying degrees of (1) customization and standardization (external perspective) on the one hand and (2) the distribution of property rights, i.e. centralization or particularization, on the other hand.

Our research contributes to the business model and business model innovation literatures as it adds two dimensions which are decisive for the value configuration especially in the sharing economy. In this respect, our study also contributes to sharing economy research. Sharing-based business models can be generated by positioning on these two dimensions.

The insights from our study also have practical implications. Firms can use the two-dimensional search grid to systematically explore opportunities for sharing-based business models. Firms not engaged in the sharing economy yet can identify market positions that might complement their regular business. Firms acting in competitive market positions in the sharing economy can examine whether shifts on these two dimensions might lead to uncontested market segments.

Despite these contributions, our research comes with limitations. The results are limited by our sample data and the case study research approach. We also do not show relations to (perceived) value distributions within the sharing network or focal firms’ performance. Instead, we introduce a new classification of antecedents of value configuration mechanisms and, therefore, provide a first concept for the strategic analysis of sharing-based business models. We encourage future studies to elaborate on the basic theories of the sharing economy and to use larger samples or archival data for further development and testing. Future research should also dig into success factors of business models in sharing networks and into the externalities that contribute to achieving a sustained competitive position. Additionally, a striking research focus should be on the question whether major crises such as the current COVID-19 pandemic (Kraus et al. 2020b) will change the sharing economy and its business models.

## Appendix

See Tables 2, 3.

**Table 2:** Customization versus completeness of property rights (case firms from Table 1)

Case firm	Customization versus standardization	Completeness of property rights
Airbnb	The content is user created without influence of Airbnb. Thus, there are no limits to the customization of the content.	Airbnb provides the execution of the transactions, property rights are decentralized.
Car2Go	The car sharing fleet consists of standardized vehicles. Since 2016, users can choose between four different models though. Availability varies between locations.	Property rights are centralized. However, organization varies between different locations, allowing for particularization.
DriveNow	The car sharing fleet consists of standardized vehicles. Users can choose between 10 models from BMW and Mini Availability varies between locations.	Property rights are centralized. Organization varies slightly between different locations, allowing for particularization.
Enterprise	The offered fleet consists of standardized vehicles. Customization possible through the diversity of vehicles.	Centralized organization, cars are not shared with users but rented to customers.
Europcar	The offered fleet consists of standardized vehicles. Customization possible through the diversity of vehicles..	Centralized organization, cars are not shared with users but rented to customers.
Flinkster	The offered fleet consists of standardized vehicles, partially with Flinkster branding. Customization possible through the diversity of vehicles and the choice between car sharing and renting.	Centralized organization, cars are not shared between users, only rented to customers in other regions.
Fon	Wi–Fi networks use standardized protocols for access and data transmission. Differences in the offering can be based on range and speed of the Wi–Fi networks.	Fon provides access to shared Wi–Fi networks. The owners of the Wi–Fi networks remain responsible for the operation.
Getaround	The car sharing fleet consists of users' private cars. Customization is possible through the diversity of offered vehicles and customizations by their owners.	Similar to Airbnb, the vehicles are privately owned and Getaround offers the web–based service to match users and providers of the vehicles. Getaround installs a special software though to allow users to locate and unlock cars.
Greenwheels	The car sharing fleet consists of three standardized vehicles.	Property rights are centralized, Greenwheels organizes the sharing processes, the network and owns the cars.
Hapimag	Hapimag's user community consists of shareholders with the right to influence the acquisition of new vacation properties. Customization is possible.	The property rights remain within the user community, decisions are reached on the board level though.
HILTI	Hilti offers a range of standardized premium tools for rent. Customization is possible through the high variety of tools.	Property rights are centralized, as Hilti remains the single owner of tools.
Lending–Club	LendingClub collects money from investing users and borrows it to other users. Customization refers to the adjustment of interest rates and credit periods.	LendingClub acts as an intermediary that allows users to invest in loans. Thus, property rights are particularized.
MyHammer	Myhammer creates a marketplace for services. Users offer or demand specific services. Thus, the content can be totally customized.	Myhammer acts as an intermediary platform that allows users to match offer and demand.
Turo	The car sharing fleet consists of standardized vehicles. Users offer their cars according to their own time and range preferences, other users rent these private cars.	Propertry rights remain with the owner of a car. Turo only organizes the sharing processes.
Sixt	The offered fleet consists of standardized vehicles. Customization possible through the diversity of vehicles	Centralized organization, cars are not shared with users but rented to customers.
TaskRabbit	TaskRabbit creates a marketplace for services. Users offer or demand specific customized services. The first one responding to a request receives the contract.	TaskRabbit matches the demand and offer of services on their platform.
Uber	Uber offers ride sharing. In contrast to car sharing, offering users drive their guests to their goal.	Property rights are peculiar. Uber only centralizes transaction processes.
Zaarly	Customization is possible on two ways. Users provide individual content and customize their sites in the network.	Property rights are peculiar. Zaarly only centralizes transaction processes.

**Table 3:** Two dimensions of value configurations in sharing-based business models

	Dimension 1	Dimension 2
Content	Customization ↔ standardization	Centralization ↔ particularization
Value configuration	Value creation	Value creation and capture
Perspective	External (customer)	Internal (sharing network)
Theoretic foundation	Generic competitive strategies of differentiation and cost leadership	Property rights theory/governance structures

.

## References

[CR1] Afuah A (2003). Redefining firm boundaries in the face of the internet: are firms really shrinking?. Acad Manag Rev.

[CR2] Akhmedova A, Marimon M-M (2020). Winning strategies for customer loyalty in the sharing economy: a mixed–methods study. J Bus Res.

[CR3] Alchian AA (1965). Some economics of property rights. Il Politico.

[CR4] Amit R, Zott C (2001). Value creation in e-business. Strateg Manag J.

[CR5] Amit R, Zott C (2010) Business model innovation: creating value in times of change. IESE Research Papers, D/870:1–15.

[CR6] Amit R, Zott C (2012). Creating value through business model innovation. MIT Sloan Manag Rev.

[CR7] Arvidsson A (2018). Value and virtue in the sharing economy. Soc Rev.

[CR8] Asher CC, Mahoney JM, Mahoney JT (2005). Towards a property rights foundation for a stakeholder theory of the firm. J Manag Gov.

[CR9] Baden-Fuller C, Mangematin V (2013). Business models: a challenging agenda. Strateg Organ.

[CR10] Bardhi F, Eckhardt GM (2012). Access–based consumption: the case of car sharing. J Consum Res.

[CR11] Barney JB (1986). Types of competition and the theory of strategy: toward an integrative framework. Acad Manag Rev.

[CR12] Barney JB (1991). Firm resources and sustained competitive advantage. J Manag.

[CR13] Belk R (2014). You are what you can access: sharing and collaborative consumption online. J Buss Res.

[CR14] Berger A, Brem A (2016). Innovation hub how–to: lessons from Silicon Valley. Glob Bus Organ Excell.

[CR15] Bouncken RB, Fredrich V (2016). Good fences make good neighbors? Directions and safeguards in alliances on business model innovation. J Bus Res.

[CR16] Bouncken RB, Kraus S, Martínez-Pérez JF (2020). Entrepreneurship of an institutional field: the emergence of coworking spaces for digital business models. Int Entrep Manag J.

[CR17] Bouncken RB, Kraus S, Roig-Tierno N (2019). Knowledge-and innovation-based business models for future growth: digitalized business models and portfolio considerations. Rev Manag Sci.

[CR18] Bouncken RB, Laudien SM, Fredrich V, Görmar L (2018). Coopetition in coworking–spaces: value creation and appropriation tensions in an entrepreneurial space. Rev Manag Sci.

[CR19] Bouncken RB, Lehmann C, Fellnhofer K (2016). The role of entrepreneurial orientation and modularity for business model innovation in service companies. Int J Entrep Ventur.

[CR20] Bouncken R, Ratzmann M, Barwinski R, Kraus S (2020). Coworking spaces: empowerment for entrepreneurship and innovation in the digital and sharing economy. J Bus Res.

[CR21] Brand M, Tiberius V, Bican PM, Brem A (2019). Agility as an innovation driver: towards an agile front–end of innovation framework. Rev Manag Sci.

[CR22] Breier M, Kallmünzer A, Clauß T, Gast J, Kraus S, Tiberius V (2021). The role of business model innovation in the hospitality industry during the COVID-19 crisis. Int J Hospital Manag.

[CR23] Brem A, Meier M, Wimschneider C (2016). Competitive advantage through innovation: the case of Nespresso. Eur J Innov Manag.

[CR24] Brettel M, Friederchsen N, Keller M, Rosenberg M (2014). How virtualization, decentralization and network building change the manufacturing landscape: an Industry 40 Perspective. Int J Inf Commun Eng.

[CR25] Cesinger B, Hughes M, Mensching H, Bouncken R, Fredrich V, Kraus S (2016). A socioemotional wealth perspective on how collaboration intensity, trust, and international market knowledge affect family firms multinationality. J World Bus.

[CR26] Chan J, Pun N, Selden M (2013). The politics of global production: apple, Foxconn and China's new working class. New Technol Work Employ.

[CR27] Cheng M (2016). Sharing economy: a review and agenda for future research. Int J Hosp Manag.

[CR28] Chesbrough H (2007). Business model innovation: it's not just about technology anymore. Strateg Leadersh.

[CR29] Chesbrough H, Rosenbloom RS (2002). The role of the business model in capturing value from innovation: evidence from Xerox Corporations technology spin-off companies. Ind Corp Change.

[CR30] Claessens S, Laeven L (2003). Financial development, property rights, and growth. J Finance.

[CR31] Clauss T, Bouncken RB, Laudien S, Kraus S (2020). Business model reconfiguration and innovation in SMEs: a mixed-method analysis from the electronics industry. Int J Innov Manag.

[CR32] Clauss T, Harengel P, Hock M (2019). The perception of value of platform-based business models in the sharing economy: determining the drivers of user loyalty. Rev Manag Sci.

[CR33] Coase RH (1960). The problem of social cost. J Law Econ.

[CR34] Cohen B, Kietzmann J (2014). Ride on! Mobility business models for the sharing economy. Organ Environ.

[CR35] Creswell J (2003). Mixed methods procedures. Research design: qualitative, quantitative, and mixed methods approaches.

[CR36] Curtis SK, Lehner M (2019). Defining the sharing economy for sustainability. Sustainability.

[CR37] Davidson A, Habibi MR, Laroche M (2018). Materialism and the sharing economy: a cross-cultural study of American and Indian consumers. J Bus Res.

[CR38] Demil B, Lecocq X, Ricart JE, Zott C (2015). Introduction to the SEJ special issue on business models: business models within the domain of strategic entrepreneurship. Strateg Entrep J.

[CR39] de Lange D, Valliere D (2020). Investor preferences between the sharing economy and incumbent firms. J Bus Res.

[CR40] Demsetz H (1967). Toward a theory of property rights. Am Econ Rev.

[CR41] Ding Y, Keh HT (2016). A re–examination of service standardization versus customization from the consumers perspective. J Serv Market.

[CR42] Eisenhardt KM (1989). Agency theory: an assessment and review. Acad Manag Rev.

[CR43] Eisenhardt KM, Graebner ME (2007). Theory building from cases: opportunities and challenges. Acad Manag J.

[CR44] Ert E, Fleischer A, Magen N (2016). Trust and reputation in the sharing economy: the role of personal photos in Airbnb. Tour Manag.

[CR45] Ferreira JJ, Fernandes CI, Kraus S (2019). Entrepreneurship research: mapping intellectual structures and research trends. Rev Manag Sci.

[CR46] Filser M, Tiberius V, Kraus S, Spitzer J, Kailer N, Bouncken R (2020). Sharing economy: a bibliometric analysis of the state of research. Int J Entrep Ventur.

[CR47] Filser M, Tiberius V, Kraus S, Zeitlhofer T, Kailer N, Müller A (2020) Opportunity recognition: conversational foundations and pathways ahead. Entrep Res J 20200124

[CR48] Foss K, Foss N (2005). Resources and transaction costs: how property rights economics furthers the resource–based view. Strateg Manag J.

[CR49] Foss NJ, Saebi T (2017). Fifteen years of research on business model innovation: how far have we come and where should we go?. J Manag.

[CR50] Fjeldstad ØD, Snow CC, Miles RE, Lettl C (2012). The architecture of collaboration. Strateg Manag J.

[CR51] Gassmann O, Enkel E, Chesbrough H (2010). The future of open innovation. R&D Manag.

[CR52] Gassmann SE, Nunkoo R, Tiberius V, Kraus S (2021) My home is your castle: forecasting the future of accommodation sharing. Int J Contemp Hospital Manag (**in press**)

[CR53] George G, Bock AJ (2011). The business model in practice and its implications for entrepreneurship research. Entrep Theory Pract.

[CR54] Geissinger A, Laurell C, Sandstrom C (2020). Digital disruption beyond Uber and Airbnb-Tracking the long tail of the sharing economy. Technol Forecast Soc Change.

[CR55] Govindan K, Shankar KM, Kannan D (2020). Achieving sustainable development goals through identifying and analyzing barriers to industrial sharing economy: a framework development. Int J Prod Econ.

[CR56] Gupta M, Esmaeilzadeh P, Uz I, Tennant VM (2019). The effects of national cultural values on individuals' intention to participate in peer-to-peer sharing economy. J Bus Res.

[CR57] Hamari J, Sjöklint M, Ukkonen A (2016). The sharing economy: why people participate in collaborative consumption. J Assoc Inf Sci Technol.

[CR58] Hartl B, Hofmann E, Kirchler E (2016). Do we need rules for “what's mine is yours”? Governance in collaborative consumption communities. J Bus Res.

[CR59] Heider A, Gerken M, van Dinther N, Hülsbeck M (2020). Business model innovation through dynamic capabilities in small and medium enterprises—evidence from the German Mittelstand. J Bus Res.

[CR60] Jandhyala S (2013). Property rights and international investment in information technology services. Strateg Manag J.

[CR61] Jia F, Li D, Liu G, Sun H, Hernandez JE (2020). Achieving loyalty for sharing economy platforms: an expectation-confirmation perspective. Int J Oper Prod Manag.

[CR62] Keay I, Metcalf C (2011). Property rights, resource access, and long–run growth. J Empir Leg Stud.

[CR63] Kraus S, Li H, Kang Q, Westhead P, Tiberius V (2020). The sharing economy: a bibliometric analysis of the state-of-the-art. Int J Entrep Behav Res.

[CR64] Kraus S, Clauß T, Breier M, Gast J, Zardini A, Tiberius V (2020). The economics of COVID–19: initial empirical evidence on how family firms in five European countries cope with the corona crisis. Int J Entrep Behav Res.

[CR65] Kraus S, Filser M, Puumalainen K, Kailer N, Thurner S (2020). Business model innovation: a systematic literature review. Int J Innov Technol Manag.

[CR66] Kraus S, Richter C, Brem A, Cheng C-F, Chang M-L (2016). Strategies for reward-based crowdfunding campaigns. J Innov Knowl.

[CR67] Laffey D (2009). Click trading: a case study of Moneynet. J Strateg Inf Syst.

[CR68] Laffey D, Gandy A (2009). Applying Stabell and Fjeldstads value configurations to E–commerce: a cross–case analysis of UK comparison websites. J Strateg Inf Syst.

[CR69] Lee J, Bagheri B, Kao H-A (2015). A cyber–physical systems architecture for industry 4.0–based manufacturing systems. Manuf Lett.

[CR70] Liguori E, Bendickson J, Solomon S, McDowell WC (2019). Development of a multi-dimensional measure for assessing entrepreneurial ecosystems. Entrep Reg Dev.

[CR71] Liu X, Chen H (2020). Sharing economy: promote its potential to sustainability by regulation. Sustainability.

[CR72] Lutz C, Newlands G (2018). Consumer segmentation within the sharing economy: the case of Airbnb. J Bus Res.

[CR73] Markides C, Charitou CD (2004). Competing with dual business models: a contingency approach. Acad Manag Executive.

[CR74] Martin CJ (2016). The sharing economy: a pathway to sustainability or a nightmarish form of neoliberal capitalism?. Ecol Econ.

[CR75] Metallo C, Agrifoglio R, Schiavone F, Mueller J (2018). Understanding business model in the Internet of Things industry. Technol Forecast Soc Change.

[CR76] Moehlmann M (2015). Collaborative consumption: determinants of satisfaction and the likelihood of using a sharing economy option again. J Consum Behav.

[CR77] Morris M, Schindehutte M, Allen J (2005). The entrepreneurs business model: toward a unified perspective. J Bus Res.

[CR78] Mumdziev N, Windsperger J (2011). The structure of decision rights in franchising networks: a property rights perspective. Entrep Theory Pract.

[CR79] Muzellec L, Ronteau S, Lambkin M (2015). Two–sided internet platforms: a business model lifecycle perspective. Ind Mark Manag.

[CR80] Oskam J, Boswijk A (2016). Airbnb: the future of networked hospitality businesses. J Tour Futures.

[CR81] Osterwalder A (2004) The business model ontology—a proposition in a design science approach, Thesis Univ. Lausanne.

[CR82] Osterwalder A, Pigneur Y (2010). Business model generation: a handbook for visionaries, game changers, and challengers.

[CR83] Osterwalder A, Pigneur Y, Tucci CL (2005). Clarifying business models: origins, present, and future of the concept. Commun AIS.

[CR84] Ostrom E (1990). Governing the commons: the evolution of institutions for collective action.

[CR85] Parente RC, Geleilate J-MG, Rong K (2018). The sharing economy globalization phenomenon: a research agenda. J Int Manag.

[CR86] Park H, Armstrong CMJ (2017). Collaborative apparel consumption in the digital sharing economy: an agenda for academic inquiry. Int J Consum Stud.

[CR87] Penrose ET (1959). The theory of the growth of the firm.

[CR88] Pies I, Hielscher S, Everding S (2020). Do hybrids impede sustainability? How semantic reorientations and governance reforms can produce and preserve sustainability in sharing business models. J Bus Res.

[CR89] Pohjola M (2002). The new economy: facts, impacts and policies. Inf Econ Policy.

[CR90] Ponce RS, Peris Cancio JA, Escámez Sánchez J (2018). The capabilities approach and values of sustainability: towards an inclusive pedagogy. J Innov Knowl.

[CR91] Porter ME (1985). Competitive advantage: creating and sustaining superior performance.

[CR92] Porter ME (1987). From competitive advantage to corporate strategy. Harvard Bus Rev.

[CR93] Porter ME (1991). Towards a dynamic theory of strategy. Strateg Manag J.

[CR94] Pouri MJ, Hilty LM (2018). Conceptualizing the digital sharing economy in the context of sustainability. Sustainability.

[CR95] Ram M, Trehan K (2009). Critical by design: enacting critical action learning in a small business context. Action Learn Res Pract.

[CR96] Rayport JF, Sviokla JJ (1995). Exploiting the virtual value chain. Harvard Bus Rev.

[CR97] Richardson J (2008). The business model: an integrative framework for strategy execution. Strateg Change.

[CR98] Richter C, Kraus S, Syrjä P (2015). The shareconomy as a precursor for digital entrepreneurship business models. Int J Entrep Small Bus.

[CR99] Schmengler K, Kraus S (2010). Entrepreneurial marketing over the internet: an explorative qualitative empirical analysis. Int J Entrep Ventur.

[CR100] Semke L-M, Tiberius V (2020). Corporate foresight and dynamic capabilities: an exploratory study. Forecasting.

[CR101] Sheth JN, Newman BI, Gross BL (1991). Why we buy what we buy: a theory of consumption values. J Bus Res.

[CR102] Spigel B (2015). The relational organization of entrepreneurial ecosystems. Entrep Theory Pract.

[CR103] Stabell CB, Fjeldstad ØD (1998). Configuring value for competitive advantage: on chains, shops, and networks. Strateg Manag J.

[CR104] Sundbo J (2002). The service economy: standardization or customization?. Serv Ind J.

[CR105] Täuscher K, Laudien SM (2018). Understanding platform business models: a mixed methods study of marketplaces. Eur Manag J.

[CR106] Teece DJ (2010). Business models, business strategy and innovation. Long Range Plann.

[CR107] Teixeira AAC, Ferreira C (2019). Intellectual property rights and the competitiveness of academic spin–offs. J Innov Knowl.

[CR108] Thompson JD (1967). Organizations in action: Social science bases of administrative theory.

[CR109] Thuong TL, Monideepa T (2009). Business ecosystem perspective on value co–creation in the Web 2.0 era: implications for entrepreneurial opportunities. Int J Entrep Ventur.

[CR110] Tiberius V (2019). Scenarios in the strategy process: a framework of affordances and constraints. Eur J Futures Res.

[CR111] Tiberius V, Schwarzer H, Roig-Dobón S (2020). Radical innovations: between established knowledge and future research opportunities. J Innov Knowl.

[CR112] Tiberius V, Siglow C, Sendra-García J (2020). Scenarios in business and management: the current stock and research opportunities. J Bus Res.

[CR113] Timmers P (1998). Business models for electronic markets. Electron Mark.

[CR114] Vidaillet B, Bousalham Y (2020). Coworking spaces as places where economic diversity can be articulated: towards a theory of syntopia. Organ.

[CR115] Wallsten S (2015) The competitive effects of the sharing economy: how is Uber changing taxis? Technology Policy Institute.

[CR116] Yin RK (2009). Case study research: Design and methods.

[CR117] Zervas G, Proserpio D, Byers JW (2017). The rise of the sharing economy: estimating the impact of Airbnb on the hotel industry. J Mark Res.

[CR118] Zott C, Amit R (2009). Designing your future business model: an activity system perspective. Long Range Plann.

[CR119] Zott C, Amit R (2010). Business model design: An activity system perspective. Long Range Plann.

[CR120] Zott C, Amit R, Massa L (2011). The business model: Recent developments and future research. J Manag.

